# Impact of intestinal dysbiosis on breast cancer metastasis and progression

**DOI:** 10.3389/fonc.2022.1037831

**Published:** 2022-11-07

**Authors:** Jin Zhang, Qiqi Xie, Xingfa Huo, Zhilin Liu, Mengting Da, Mingxue Yuan, Yi Zhao, Guoshuang Shen

**Affiliations:** Affiliated Hospital of Qinghai University, Affiliated Cancer Hospital of Qinghai University, Xining, China

**Keywords:** gastrointestinal flora, intestinal dysbiosis, breast cancer, metastasis, progression

## Abstract

Breast cancer has a high mortality rate among malignant tumors, with metastases identified as the main cause of the high mortality. Dysbiosis of the gut microbiota has become a key factor in the development, treatment, and prognosis of breast cancer. The many microorganisms that make up the gut flora have a symbiotic relationship with their host and, through the regulation of host immune responses and metabolic pathways, are involved in important physiologic activities in the human body, posing a significant risk to health. In this review, we build on the interactions between breast tissue (including tumor tissue, tissue adjacent to the tumor, and samples from healthy women) and the microbiota, then explore factors associated with metastatic breast cancer and dysbiosis of the gut flora from multiple perspectives, including enterotoxigenic Bacteroides fragilis, antibiotic use, changes in gut microbial metabolites, changes in the balance of the probiotic environment and diet. These factors highlight the existence of a complex relationship between host-breast cancer progression-gut flora. Suggesting that gut flora dysbiosis may be a host-intrinsic factor affecting breast cancer metastasis and progression not only informs our understanding of the role of microbiota dysbiosis in breast cancer development and metastasis, but also the importance of balancing gut flora dysbiosis and clinical practice.

## 1 Background

### 1.1 Epidemiology and staging of breast cancer

The 2020 Global Cancer Statistics report shows that female breast cancer is the most common cancer worldwide, with the highest number of new cases annually (approximately 11.7% of all new cases in both men and women), having overtaken lung cancer (11.4%) (1). There are four main subtypes of breast cancer, approximately 75% of them are positive for ER and/or PR (2). The luminal A (ER and PR positive, HER2 negative, low Ki67) subtype accounts for approximately 40% of all cases; it is characterized by low invasiveness, a low recurrence rate, a high survival rate, and the best response to hormonal therapy (3). In turn, the luminal B (ER and PR positive, HER2 positive or HER2 negative, with high Ki67) subtype is responsible for 10–20% of all cancer cases, has a higher relapse rate, proliferative index, and lower recurrence survival (4–6). HER2 positive (non-luminal) were defined as HER2 overexpression or amplification, ER and PR absence, and survival rate significantly improvement with targeted therapy (7). Triple‐negative breast tumors (TNBC) are defined as ER, PR, and HER2 negative. TNBC which makes up approximately 15% of all breast tumors and have a high risk of distant relapse in the first 3 to 5 years following diagnosis (8, 9).

With advances in early diagnosis and comprehensive treatment, the prognosis for patients with breast cancer has improved; however, the incidence of metastasis is also increasing (10). It has been reported that 20%–30% of patients with breast cancer can develop metastases after diagnosis and treatment of the primary tumor, with metastases being the cause of approximately 90% of deaths (11). Breast cancer shows a tendency to metastasize to a variety of organs, including bone, lung, liver, and brain, which is termed metastatic heterogeneity. Bone metastases account for approximately 75% of metastases (12), with an overall 5-year survival rate of 22.8% (13). Lung is the second most common site of breast cancer metastasis (14), with an overall 5-year survival rate of 16.8%. The liver is second only to lung as a metastasis site, but survival is poor relative to local, bone, and lung recurrences, with an expected 5-year overall survival rate of 8.5% (15). Brain accounts for approximately 15%–30% of metastatic sites in patients with metastatic breast cancer, limiting quality of life and a very short life expectancy (16–18).

The priority of metastasis varies from organ to organ, resulting in differences in prognosis and treatment response. A widely accepted model of metastasis is the “seed and soil” hypothesis proposed by Paget (19), which initially revealed that successful colonization of second organs depends on the intrinsic properties of the tumor cells and the compatibility and support of the microenvironment.

### 1.2 Intestinal flora dysbiosis

#### 1.2.1 Gut microbiota composition in human health

A dynamic balance is maintained between the microbiota and the host, and this balance plays an important role in human health by influencing the physiological functions of the organism. A healthy intestinal microbiota is composed mainly of the *phyla Firmicutes* and *Bacteroidetes*, followed by *the phyla Actinobacteria* and *Verrucomicrobia* (20). The distribution of microorganisms in the gastrointestinal tract varies longitudinally from the esophagus to the rectum, Helicobacter is the dominant species in the stomach and determines the microbial status of the entire gastric flora. While H. pylori inhabits the stomach as a commensal, other genera constitute the rich diversity of the gastric flora (21, 22). Conversely, this diversity is reduced when *H. pylori* cause disease. *Firmicutes* and *Actinobacteria* are the most dominant phylum in the duodenum (23). The jejunum is dominated by the growth of Gram-positive aerobic and facultative anaerobes, including *Lactobacilli, Enterococci* and *Streptococci.* In the ileum, with predominance of aerobic species, while the distal ileum has a similar bacterial body to the colon, with anaerobes and Gram-negative organisms (23). The bacteriophage in the large intestine is dominated by *the phyla Firmicutes* and *Bacteroidetes*. Furthermore, there are other important pathogens in the human colon, such as *Campylobacter jejuni*, *Salmonella enterica*, *Vibrio cholera and Escherichia coli (E. coli)*, and *Bacteroides fragilis* (24, 25). The abundance of the Proteobacteria phylum is significantly lower in normal humans, and its absence along with the high abundance of genera such as *Bacteroides*, *Prevotella* and *Ruminococcus* indicates a healthy gut microbiota (26).

#### 1.2.2 Gut microbiota function

Intestinal flora homeostasis has an important role in maintaining normal body function, The gut microflora creates a stable mucosal barrier for the intestine to prevent the invasion of pathogenic microorganisms (27). Gut microbes break down non-digestible compounds through anaerobic fermentation to produce compounds of short-chain fatty acids (SCFAs), which have good anti-inflammatory and chemopreventive properties and act as barrier protectors (28, 29) and are considered as tumor suppressors (30). Microorganisms containing Lipopolysaccharide (LPS), such as Salmonella and Escherichia coli, activate antigen presenting cells through pattern recognition receptors to produce cytokines, which together with endogenous glycolipid antigens and the major histocompatibility complex (MHC) class I-related glycoprotein CD1d activate Invariant natural killer T (iNKT) cells and participate in various immunomodulatory responses (31, 32). In addition, many intestinal microbiota are involved in bone remodeling processes as immunomodulators, such as *Lactobacillus acidophilus*, *Lactobacillus plantarum*, *Lactobacillus rhamnosus GG*, *Lactobacillus reuteri*, *Lactobacillus paracasei* and *Bacillus clausii* (33). The gut microbiota regulates nutrient metabolism by regulating lipid metabolism, propionic acid in short-chain fatty acids reduces fatty acid levels in liver and plasma and reduces food intake (34), and the gut microbiota regulates intestinal and plasma Lipopolysaccharide (LPS) levels by modulating the intestinal endocannabinoid (eCB) system (35), which affects adipose tissue metabolism. Intestinal flora are involved in the production of gastrin, insulin (36) and glucagon-like peptide-1 (GLP-1) (37, 38) through a paracrine pathway produced by enterocytes, and it is also involved in the synthesis of bile acids, cholesterol, bound fatty acids (39) and vitamin (40), thereby regulation of endocrine levels and metabolic changes in the host. The gut microbiota synthesizes a number of neurochemicals, (e.g., gamma amino butyric acid (GABA): an inhibitory neurotransmitter), which influence central nervous and gut function (41). A gut-brain microbial axis exists between gut microbes, the gastrointestinal tract and the central nervous system (42), which links brain emotional centers to mechanisms such as gut function, gut neural reflexes and gut endocrine signaling to jointly coordinate organismal changes (43, 44). Circulating SCFAs produced by gut microbiota metabolism affect the integrity of the blood-brain barrier (BBB) by increasing the production of tight junction proteins, and increased BBB integrity reduces the entry of undesirable metabolites into brain tissue and strengthens the defense mechanisms of the blood-brain barrier (45). Compounds produced by the metabolism of the gut microbiota, such as lipoproteins and lipopolysaccharides, affect autoimmune function by stimulating the release of cytokines from immune cells. These cytokines can cross the BBB and activate neurons, altering neurological function and leading to changes in mood and behavior (46), providing new ideas for the treatment of brain dysfunction.

#### 1.2.3 Dysbiosis

Dysbiosis refers to a state in which the intestinal flora loses its normal “beneficial” function and is continuously disturbed, causing disease. It is associated with a large proportional change in the composition of the microbiota beyond the normal range caused by host-related and environmental factors (47). Dysbiosis is usually characterized by the following feature: Bloom of pathobionts (48), Loss of commensals (49) and Loss of alpha diversity (50–52), which can be present individually or simultaneously and mutually exclusive. Currently, dysbiosis has a causal relationship with the manifestation, diagnosis or treatment of specific diseases, from the perspective of the composition of the intestinal microflora, mainly originating from Infection and inflammation (53, 54); Diet and xenobiotics (55, 56); Genetics (57) and Familial transmission (58–60) etc.

#### 1.2.4 Link between dysbiosis and cancer

Dysbiosis states may negatively affect the organism leading to various disease states. The microbiota may have some tumor suppressive effects on the host, and deviations in flora balance may be associated with cancer development (61). Studies (62–67) have identified direct and indirect roles of the gut microbiota in carcinogenesis, cancer treatment and prevention. including colon (66, 68, 69), gastric (70–73), lung (74, 75), prostate (76–78) and breast cancers (79) (**Tables 1**, **2**), and suggest that the gut microbiota and these cancers are interlinked through tumor suppression and tumor initiation factors. Modification of the composition and activity of the intestinal flora through the administration of prebiotics, probiotics and synthetics, providing benefits to patients with colorectal cancer, such as: modulation of immunity, improvement of bile acid metabolism and restoration of intestinal microbial diversity (68). *H. pylori* is one of the major causative factors of gastric cancer. Probiotics against *H. pylori* through various mechanisms, including: secretion of antibacterial compounds; inhibition of *H. pylori* colonization; action through stimulation of mucin synthesis; and modulation of host immune response, which provides new perspectives on gastric cancer prevention and treatment (100). It was found that memory T and NK cell profiles were increased in peripheral blood samples from patients with beneficial and diversity-rich gut microbes. This has important implications for predicting the response to anti-PD-1 immunotherapy in Chinese non-small-cell lung cancer patients (101). Gram-positive bacteria stimulate the production of specific subpopulations of “pathogenic” T helper 17 (pTh17) cells and memory Th1 immune responses, and the absence of these bacteria leads to reduced pTh17 responses and cyclophosphamide tumor resistance, demonstrating that the gut microbiota contributes to the formation of anti-cancer immune responses in lung cancer patients (102). However, the symbiotic gut microbiota promotes endocrine resistance in castration-resistant prostate cancer by providing an alternative source of androgens, implying that the gut flora may play a negative role in this process (103). Gut bacteria can regulate insulin-like growth factor-1 (IGF1) levels in the host *via* short-chain fatty acids, thereby promoting the proliferation of prostate cancer cells, then modulating the gut microbiota to influence the gut microbiota-IGF1-prostate axis may be beneficial in the prevention and treatment of prostate cancer (104). In addition, the use of gut microbiota analysis to predict patient response to immune check inhibition sites has emerged in cancer treatment, e.g., breast cancer (105). Currently, the role of gut microbes in the development of various cancers varies, and their variation may have implications for achieving more personalized precision medicine in oncology.

**Table 1:** A summary of studies addressing changes in microbiota between breast cancer tissue, non-cancerous adjacent tissue and healthy breast tissue.

REF	Mian methodology	Sample type	Microbiome related results		
			Normal breast tissue	Non-cancerous adjacent tissues	Breast cancer (BC)
(80)	Pyrosequencing V4 16S rDNA Pipeline: QIIME	20 BC patients		↑*Sphingomonas yanoikuyae*	↑*Methylobacterium radiotolerans*
(81)	V3-V4 16S rRNA sequencing (Illumina) Pipeline: UCLUST	57 women with invasive breast carcinoma and 21 healthy women	↑*Methylobacterium*		↑*Alcaligenacea*
(82)	V3-V5 16S rRNA amplified sequencing data	668 tumor tissues and 72 normal adjacent tissues from The Cancer Genome Atlas (TCGA)		↑*Actinobacteria and Firmicutes*	↑*Proteobacteria*, *Mycobacterium fortuitum* and *Mycobacterium phlei*
(83)	V1-V2 16S rRNA sequencing (Illumina HiSeq)	22 Chinese patients with benign tumor and 72 malignant BC patients			↑*Propionicimonas, Micrococcaceae, Caulobacteraceae, Rhodobacteraceae, Nocardioidaceae* and *Methylobacteriaceae *(Ethnicity-related); ↓*Bacteroidaceae* and ↑ *Agrococcus* (with malignancy)
(84)	Pathochips array	20 normal breast tissue and 148 BC tissue			↑*Actinomyces, Aerococcus, Arcanobacterium, Bifidobacterium, Bordetella, Cardiobacterium, Corynebacterium, Eikenella, Fusobacterium, Geobacillus, Helicobacter, Kingella, Orientia, Pasteurella, Peptinophilus, Prevotella, Rothia, Salmonella, and Treponema*
(85)	Pathochips array	100 women with triple negative BC (TNBC), 17 matched controls and 20 non-matched controls			↑*Arcanobacterium* (75%), *Brevundimonas*, *Sphingobacteria, Providencia*, *Prevotella*, *Brucella, Eschherichia*, *Actinomyces, Mobiluncus*, *Propiniobacteria, Geobacillus*, *Rothia, Peptinophilus*, and *Capnocytophaga* (Canimorsus) ↑*Herpesviridae*, *Retroviridae, Parapoxviridae*, *Polyomaviridae, Papillomaviridae *(virus)
(86)	V3 16S-rRNA gene amplicons sequencing (Ion Torrent)	16 Mediterranean patients with BC	↓*Methylobacterium* (↑*Ralstonia)*		↑*Sphingomonas*
(87)	V6 16S rRNA gene sequencing (Illumina MiSeq) Pipeline: QIIME	58 women after surgery:13 benign, 45 cancerous tumors and 23 healthy women	↓ *Prevotella*, *Lactococcus*, *Streptococcus, Corynebacterium and Staphylococcus*		↑*Bacillus, Staphylococcus*, *Enterobacteriaceae * (unclassified)*, Comamondaceae *(unclassified) *and Bacteroidetes *(unclassified)
(88)	V3-V5 16S rDNA hypervariable taq sequencing (Illumina MiSeq) Pipeline: IM-TORNADO	28 women undergoing non-mastectomy breast surgery: 13 benign breast disease and 15 invasive BC			↓*Fusobacterium*, *Atopobium, Gluconacetobacter*, *Hydrogenophaga* and *Lactobacillus*
(89)	V4 16S rRNA gene sequencing (Illumina MiSeq) Pipeline: Mothur	25 women with breast cancer and 23 healthy women	↓unclassified genus of the *Sphingomonadaceae* family in NAF		↑*Alistipes*
(90)	V4 16S rRNA gene sequencing (Illumina Miseq)	32 women with BC stage 0 to II			↓*Akkermansia muciniphila* (AM) in BC patients with elevated body fat.
(91)	V3-V4 and V7-V9 16S rRNA gene sequencing	221 patients with breast cancer, 18 individuals predisposed to breast cancer, and 69 controls.	↑*Stenotrophomonas* and *Caulobacter*		↓*Propionibacterium* and *Staphylococcus*
(92)	16s rRNA gene sequencing;Quantitative Insights into Microbial Ecology (QIIME) tool;RStudio	Bilateral normal breast tissue samples (n = 36) and breast tumor samples (n = 10)	↑(OUT)[*Mogibacteriaceae*] family, and *Flavobacterium*, *Acinetobacter*, and *Brevibacillus* genera		↑(OUT) *Ruminococcaceae*, *Rikenellaceae*, genera *Butyricimonas*, *Sutterella*, and *Akkermansia*.
(93)	Kraken2 and Metaphlan3	breast tumours and normal tissues (from cancer-free women) of 23 individuals (Slovak); 91 samples obtained from SRA database (China)	↑*Proteobacteria* (47%), *Bacteroidetes*, *Firmicutes and Actinobacteria* (12%)(Slovak women); ↑*Proteobacteria *(42%), *Firmicutes*(42%), *Actinobacteria* (5%), *Cyanobacteria* (4%)		↑*Acinetobacter*, *Rhodobacter, Micrococcus*, order *Corynebacteriales* and *Priestia megaterium* (Slovak patients) ↑*Streptomyces*, *viruses Siphoviridea* and *Myoviridae* (China patient)
(94)	Illumina MiSeq sequencing	Tumor tissue and normal tissue in 34 women	↑*Actinobacteria*, ↓*Proteobacteria*,		↑*Firmicutes* and *Alpha-proteobacteria*

↑ means up, ↓ means down.

**Table 2:** A summary of studies addressing changes in gut flora between breast cancer patients and non-breast cancer patients.

REF	Mian methodology	Sample type	Gut flora related results	
			Non-breast cancer patients	Breast cancer patients
(95)	Real-time qPCR targeting specific 16S rRNA sequences	31 women with early-stage BC: 15 stage 0, 7 stage I, 7 stage II and 2 stage III.		↑*Bacteroidetes*, *Clostridium coccoides* cluster, *Clostridium leptum* cluster, *Faecalibacterium prausnitzii*, and *Blautia* spp. in patients with stage II/III BC compared to patients in stage 0/I.
(96)	V3-V4 16S rRNA sequencing (Illumina) Pipeline: QIIME	48 postmenopausal women with BC and 48 paired control women		↑C*lostridiaceae*, *Faecalibacterium*, and *Ruminococcaceae* and ↓ *Dorea* and *Lachnospiraceae* in BC patients compared to controls.
(97)	Illumina sequencing	18 premenopausal BC patients, 25 premenopausal healthy patients, 44 postmenopausal BC patients and 46 postmenopausal healthy patients		↑*Escherichia coli*, *Citrobacter koseri, Acinetobacter radioresisten*s, *Enterococcus gallinarum, Shewanella putrefaciens, Erwinia amylovora, Actinomyces* spp. *HPA0247*, *Salmonella enterica*, and *Fusobacterium nucleatum* and ↓*Eubacterium eligens* and *Roseburia inulinivorans* in postmenopausal BC patients.
(98)	16s rRNA gene sequencing	54 premenopausal women with breast cancer and 28 normal premenopausal women	↑*Photobacterium*, *Pseudobutyrivibrio*	↑*Firmicutes/Bacteroidetes* (F/B) Ratio; ↑*Parasutterella* and *Campylobacter*
(99)	V3–V4 16S rRNA Gene Sequencing	30 healthy women controls and 25 breast cancer patients	↑*Bacteroidetes*	↑*Firmicutes*

↑ means up, ↓ means down.

### 1.3 The role of microbiota in breast tumourigenesis

#### 1.3.1 Estrogen and metabolism

The gastrointestinal microbiome regulates systemic estrogen, and the development of postmenopausal breast cancer is associated with disordered (high) levels of estrogen in the body (106). The metabolism of estrogen occurs in the liver, where the metabolites are conjugated and excreted into the gastrointestinal lumen within the bile. They are de-conjugated by β-glucuronidase-producing bacteria in gastrointestinal lumen, and then they are reabsorbed as free estrogens through the enterohepatic circulation to reach breast (107). All the genes in the gut flora that metabolize estrogen are collectively known as the estrobolome (108). A study found that difference s in urinary estrogen levels were associated with beta-glucuronidase activity in pre- and post-menopausal women, and that gastrointestinal flora could influence non-ovarian estrogen levels *via* the enterohepatic circulation (109). In addition, urinary estrogen levels in men and postmenopausal women were strongly correlated with all indicators of microbiota richness and diversity in faeces, with non-ovarian-acting systemic estrogens significantly associated with fecal Clostridium perfringens (including non-clostridial and three genera of *the family Rhizobiaceae*), and Gut microbiota may influence estrogen-related diseases in the elderly (109), such as Postmenopausal Breast Cancer. Many of the microbes associated with breast cancer have the β-glucuronidase enzymatic activity mentioned above, which prevents the binding of estrogen and other compounds and makes them biologically active, thus affecting local and systemic levels of estrogen and its metabolites (79, 110). During estrogen metabolism, the gut acts as an important site for estrogen reactivation and microorganisms act locally or distally to regulate disease development and homeostasis (111). When the balance of the intestinal environment is disrupted and the structure and ratio of the flora are imbalanced, excess intestinal bacteria, Lipopolysaccharides and pro-inflammatory cytokines are produced, and this change disrupts the integrity of the intestinal mucosa, which in turn triggers inflammation after bacterial translocation (112). In addition to those involving hormone metabolism (estrogen and progesterone),Studies in growing numbers are exploring the relationship between the gut microbiome and breast cancer risk *via* a non-estrogen-dependent pathway. Obesity, insulin resistance, dyslipidemia, leukocytosis, and elevated C-reactive protein (113) are associated with reduced gut microbial diversity, some of which are associated with breast cancer. Studies have demonstrated that metabolic health status (as defined by the homeostasis model assessment of insulin resistance [HOMA-IR] index, or fasting insulin level), but not obesity per se, may be an associated factor in the risk of postmenopausal breast cancer development, suggesting that hyper insulinemia is an important risk factor for breast cancer (114). Karen L Margolis et al. demonstrated an increased risk of invasive breast cancer in postmenopausal women with higher white blood cell counts (115), Nicholas J Ollberding et al. concluded that circulating C-reactive protein levels44 reflecting adipokines and systemic inflammation were associated with the risk of postmenopausal breast cancer, independent of Body fat rate (116), These further support the possibility that inflammation may be associated with the initiation, promotion and progression of breast cancer. In addition, breast cancer in postmenopausal women is significantly associated with the immune-recognised (IgA-positive) and -unrecognised (IgA-negative) gut microbiota, the former possibly through immune-mediated pathways and the latter possibly through the enterohepatic circulation effects of estrogen (117). It was shown that the microbiota of breast tissue is different from that of mammary skin tissue, where bacterial species are more abundant than in skin tissue, and more operative taxonomic units (mostly low abundance) were observed in the breast tissue microbiota. These taxa with different abundance were from *the phyla Firmicutes*, *Actinobacteria*, *Bacteroidetes*, and *Proteobacteria* (88). A comparison of breast tissue from breast cancer patients and normal women revealed higher levels of Enterobacteriaceae and Staphylococcus and increased numbers of Bacillus in breast cancer patients (87). In contrast, Lactobacillus and Streptococcus were higher in healthy women and have anticancer properties that may play a role in the prevention of breast cancer (118). *Prevotella*, which produces SCFAs propionic acid and exerts benefits in the intestine, was higher in healthy women compared with breast cancer patients (119). Further study of bacterial metabolites and bacterially induced host metabolites would provide insight into the role of bacteria in the role of breast disease will provide important information.

#### 1.3.2 The role of antibiotics

Indirect evidence suggests that the development of breast cancer is strongly associated with alterations in specific microbiota when taking antibiotics or probiotics. Through a large-scale analysis of nearly 4 million women, Simin et al. (120) showed a specific dose-dependent relationship between antibiotic use and breast cancer, with a different correlation between the type of antibiotic and breast cancer risk, such as β-lactams, macrolide (121). Irregular use or overuse of antibiotics may increase the risk of gut dysbiosis and decrease microbial diversity, and this effect may be long-lasting (122, 123), For example: co-amoxiclav and clarithromycin, Cefprozil, Amoxicillin, etc. Also, overuse of antibiotics (*penicillins*, *streptomycin*, *chloramphenicol*, *tetracyclines*, *erythromycin*, *cephalosporins* and their analogues) decreases plasma levels of lignans-enterolactone, which can increase the risk of breast cancer by affecting the microbiota (124). A study has shown that the increased excretion of bound estrogens in the feces of patients treated with ampicillin suggests that the gut microbiota are actively involved in estrogen metabolism and can have some effect on the pathogenesis of breast cancer by altering the individual’s microbial status (106). Antibiotics have been shown to disrupt the microbiota, leading to a reduced response by tumor cells to platinum-based chemotherapy and immunotherapy (106, 125, 126), suggesting that a stable microbiota is necessary for an optimal response to antitumor therapy.

#### 1.3.3 Regulation of chronic inflammation and immunity

Microbiota may promote the risk of malignancy by inducing the persistence of chronic inflammation, disrupting the balance between cell proliferation and death in the body, and triggering uncontrolled innate and adaptive immune responses (127, 128). A putative inflammatory mechanism associated with breast carcinogenesis has been demonstrated to be the upregulation of cyclooxygenase 2 (COX2) and its product prostaglandin E2 (PGE), which would increase the expression of aromatase in adipose tissue, thereby promoting the conversion of androgen precursors to estrogens (129, 130) and increasing the risk of breast carcinogenesis. Studies have demonstrated that a potential inflammatory biomarker, mucosal secretory immunoglobulin A (IgA) (131), can maintain the integrity of the mucosal barrier by regulating the composition of the intestinal microbial community, thereby attenuating the host’s innate immune response. The link between breast cancer and the mucosal secretory IgA has been established (117). This mechanism places some limits on the participation of intestinal microbial antigens in the circulation of the body, and some limits on the invasiveness of potentially dangerous microorganisms (132). Certain specific microbiota may also maintain breast health by stimulating the host inflammatory response. For example, specific bacteria S. yanoikuyae are present in the breast tissue of healthy women and their abundance is significantly reduced in the corresponding tumor tissue. An increase in its abundance may lead to a decrease in bacterial-dependent immune cell stimulation in the body, resulting in a reduced environmental risk level for the development of breast tumors (80). Studies have also confirmed the role of microorganisms in regulating specific immune processes in the development of cancer (133), For example, *Lactococcus* spp. can activate important cells associated with tumor growth (murine splenic NK cells), maintain their cytotoxicity, and enhance cellular immunity (134). In another case-control study, Goedert and colleagues (117) investigated the role of immunity and inflammation in breast cancer risk and whether the gut microbiota differed in the composition of the immune recognition microbiota and found significant differences in the composition, abundance and alpha diversity of the microbiota between the IgA+ and control IgA- groups in cancer cases and correlated with changes in high and low estrogen levels. This suggests a significant association with IgA+ and IgA- gut microbiota in postmenopausal women with breast cancer, suggesting that the gut microbiota may influence breast cancer risk through altered metabolism, estrogen cycling and immune pathways.

#### 1.3.4 Genomic stability and DNA damage

DNA damage may not be sufficient to promote cancer development, but microbes can trigger transformation by destabilizing genes, cell proliferation and death, and it has been demonstrated that microbes cause cancer development by damaging host DNA in order to survive (135). Urbaniak et al. (87) found that *Escherichia coli* (a member of the *Enterobacteriaceae family*) isolates and a *Staphylococcus* epidermidis isolate from normal adjacent tissues of breast cancer patients had the ability to induce DNA double-strand breaks, thus causing genomic instability (136). In addition, some bacterial species may eventually lead to genotoxicity by increasing the production of reactive oxygen species (137).

## 2 Current status on breast cancer progression, metastasis and microbiology

### 2.1 Enterotoxigenic bacteroides fragilis

Bacteroides fragilis is a common colonic colonizing enterobacterium (138) whose virulence is attributed to a 20 kDa zinc metalloprotease toxin known as B. fragilis toxin (BFT) (139). With reference to the effect of enterotoxigenic B. fragilis (ETBF) intestinal or ductal colonization on breast cancer progression in the mammary intraductal model, Parida S et al. (140) colonized BALB/c mice *via* the teats with ETBF or a non-toxic mutant B. fragilis (086Mut) that does not secrete BFT. The presence of BFT was found to be detected in the mammary glands of ETBF-carrying mice compared to controls, with a 3.9-fold higher tumor volume than 086Mut controls, enhanced lung and liver metastases, and more proliferative tumors forming in the ETBF group, exhibiting a mesenchymal phenotype. Moreover, trichrome staining showing significantly higher stromal infiltration, demonstrating that ETBF intestinal or ductal colonization was associated with breast cancer progression and distant metastasis. Furthermore, significant differences in breast tissue structure were found in the ETBF group compared with the 086Mut control group (140), including extensive local inflammation and tissue fibrosis, Ki-67 and proliferating cell nuclear antigen staining showed increased epithelial cell proliferation, CD3 staining showed increased T-cell infiltration, and significantly altered expression of pan-keratin, all indicating that BFT was associated with a significant increase in oncogenic cell activity and growth rate. The study also found that RNA-seq analysis of secondary tumors arising from breast cancer cells treated with BFT showed enrichment of the β-catenin pathway. The expression of several Notch-responsive genes was enriched in breast cancer cells suggesting that BFT also triggered activation of the Notch1 pathway. The results advance our understanding of the molecular mechanisms associated with ETBF/BFT and breast cancer progression (140), and point to a hypothesis that dysbiosis or disruption of the gut flora might be associated with breast cancer metastasis and progression, and that inhibition of manipulable key molecules or pathways could potentially reduce the impact of ETBF infection on breast cancer.

In looking at whether BFT affects the tumorigenicity of breast cancer cells, the team found that, compared with cells from the control group, BFT-pretreated MCF-7 and MCF-10A cell groups showed greater invasion and migration, with local tumor expansion and the formation of multifocal tumors resembling local metastases (140).However, it was not clear whether ETBF spread from within the gut to the breast or whether gut-infected mice acquired the mammary gland infection through environmental factors. Data from RNA-seq analysis of secondary tumors with limited *in vivo* formation showed higher expression of genes associated with migration, homing, and metastasis in the BFT pre-treatment group, suggesting that BFT production by ETBF intestinal colonization might be associated with the initiation of breast cancer metastasis; and breast cells exposed to BFT showed dramatic changes supporting cell motility, embryonic pluripotency pathways, expression of metastatic genes, and molecular mechanisms. However, it cannot be demonstrated that ETBF can be the sole driver directly triggering the transformation of human breast cells into tumor cells or interacting with other microbiota to show oncogenic activity.

### 2.2 Antibiotic-induced intestinal flora dysbiosis and the progression of breast cancer metastasis

To assess the effect of pre-established dysbiosis on the metastasis of hormone receptor–positive (HR+) breast cancer in a more aggressive and metastatic tumor model, Parida S et al. (141) evaluated tumor spread to the lung and axillary lymph nodes in a highly metastatic MMTV-PyMT mouse model with reference to the poorly metastatic HR+ mouse breast cancer cell line BRPKp110. The results were similar to those observed in the BRPKp110 cell line: where the spread of tumor cells to the lung was significantly increased after commensal dysregulated of the intestinal flora due to antibiotic treatment, independent of tumor volume. Moreover, the tumors progressed with the same kinetics regardless of the symbiotic dysregulation status in the experimental mice, suggesting that symbiotic dysregulation has a significant and sustained effect on HR+ breast cancer dissemination and that the enhanced ability of cancer cells to spread in symbiotically dysregulated mice is independent of tumor growth kinetics. To confirm the impact of the flora-dysregulation-driven host-intrinsic differences in inducing propagation in a mammary tumor model, they tested the symbiotic dysregulation using the L-Stop-L-KRasG12Dp53flx/flxL-Stop-L-Myristoylated p110α-GFP+ induced mouse model of breast cancer (141), and found that consistent with that observed in the homozygous model, the lungs of mice with dysbiosis of the intestinal flora showed a higher frequency of disseminated tumor cells. No significant increase in GFP+ tumor cells was observed in the distal lymph nodes. Those results confirmed that dysbiosis is independent of primary tumor growth and is associated with enhanced tumor cell dissemination; they also suggest that the tumor dissemination enhancement is the result of host dysbiosis rather than of intrinsic differences in tumor aggressiveness. Macrophages in the mammary gland may promote the metastasis of mammary tumors in experimental animals (142). Parida S et al. (141) found that commensal dysbiosis influenced the frequencies and numbers of macrophages during early or advanced stages of mammary tumor progression. Macrophages are one of the most abundant cell types within the breast tumor-microenvironment (143) and are a significant prognostic indicator of reduced survival for patients diagnosed with HR+ breast cancer (144). They observed that the majority of myeloid infiltrates within the mammary tumor microenvironment were M2-like macrophages during at early and advanced stages of tumor progression based upon CD206 expression. Importantly, the number of infiltrating tumor-promoting M2-like macrophages was significantly increased in advanced tumors of mice in the dysbiotic mice compared to non-dysbiotic controls with equal tumor burden. These data suggest that systemic expression of inflammatory mediators is increased in mice with dysbiosis tumors and that commensal dysbiosis acts synergistically with developing tumors to enhance myeloid recruitment into mammary tumors. Enhanced interstitial density or dense breast tissue is a recognized risk factor for the development of breast cancer metastases (145) and intra-mammary pro-tumor inflammation (146). They found that pre-established dysbiosis was associated with significantly enhanced collagen deposition in normal adjacent mammary glands and in tumors, and that collagen accumulation was slightly increased in the lungs of advanced tumor-bearing mice with dysbiosis, suggests that enhanced local and distal fibrosis is a long-term consequence of dysbiosis during breast cancer. Parida S et al. to determine whether gastrointestinal dysbiosis is sufficient to enhance mammary tumor cell dissemination (141), and a fecal microbiota transplantation (FMT) method was used, Both the experimental and control group and control groups were BRPKp110 breast tumor cells, Mice receiving flora-dysregulated cecal contents by FMT also showed enhanced infiltration of inflammatory myeloid cells into the mammary tissue and increased accumulation of myeloid cells into tumor tissue. Similar effects were observed in the mammary gland and tumor tissue during the advanced stages of tumor progression—that is, mammary gland tissue and tumors showed enhanced tissue fibrosis. Importantly, the spread of tumor cells to peripheral blood, lung, and distal axillary lymph nodes was also significantly increased in mice receiving dysbiosis flora (rather than “normal” FMT) by FMT, considering that a dynamically imbalanced microbiome is sufficient to enhance the metastatic spread of breast cancer. Moreover, it may be an independent correlate of the distant spread of tumor cells. Further supporting the idea that dysbiosis contributes to the evolution of breast tissue and/or tumors toward more aggressive and high-grade disease. regardless of the metastatic potential of the HR+ breast tumor model used in the study, dysbiosis of the gut flora was associated with enhanced dissemination and metastasis of breast tumor cells.

Changes in the gut microbiota also to effects in metabolites, and inflammatory signaling pathways can be amplified or inhibited. Using an *in-situ* mouse model of breast cancer, Kirkup et al. (147, 148) found significant differences in metabolic regulatory pathways across the tumor transcriptome in animals treated with broad-spectrum antibiotics, and single-cell transcriptomics revealed that the stromal cell population was altered in breast tumors from antibiotic-treated mice. The main form of the alteration was an increased number of mast cells, which accelerate tumor progression. The breast cancer model used a PyMT-derived ductal lumen cell line (PyMT-BO1) to investigate the role of gut microbiota in regulating the growth of primary mammary tumors (149). Disruption of intestinal microbiota by gavage administration of oral antibiotics (vancomycin, neomycin, metronidazole, amphotericin, and ampicillin [VNMAA]) prior to administration of tumor cells to animals, producing severe intestinal microbial changes (150, 151), and although no significant differences in tumor tissue structure were observed in those animals compared with a control group receiving plain water, significantly accelerated tumor growth was observed. Under a similar treatment regimen (152), enhanced growth resembling basal-like breast cancers was observed when spontaneously derived basal cells (EO771) were implanted *in situ*, suggesting that antibiotic-induced microbiota disruption can drive disease progression in multiple breast cancer subtypes. To determine the effect of the VNMAA mixture on the microbiota, microbial DNA was isolated from the cecum of control and VNMAA-treated animals on day 18 and subjected to birdshot macro-genomics analysis. The analysis revealed dramatic changes in the populations and overall diversity of the bacteria obtained from the animals that received VNMAA treatment, with the Shannon diversity index showing that the abundance of several microbial communities in the gut of antibiotic-treated mice was significantly reduced. In parallel, some communities (e.g., *Fusobacterium nucleatum*) persisted or overgrew. The composition of the gut microbiota was significantly altered in terms of species, abundance and overall diversity following the use of antibiotics, which was associated with accelerated tumor growth and an increase in mast cells in the tumor stroma. To determine whether mast cells affected tumor growth, Kirkup et al. (147) treated control and VNMAA-treated tumor-bearing mice with cromolyn (a mast cell stabilizer) and found that cromolyn inhibited tumor growth in the antibiotic-treated animals. Notably, the VNMAA-treated group without cromolyn treatment showed a significant increase in tumor size when compared to the control animals treated with cromolyn, and an increase in the number of mast cells was observed in sections of the EO771 tumor stroma taken from VNMAA-treated mice (147, 148). Those data suggessed the key role that mast cells play in tumor progression after antibiotic-induced microbiota disruption in mouse breast cancer: when vancomycin alone was used to induce microbiota disruption, effects similar to those already described were observed in a completely different model of breast cancer. Possibly, microbiota disruption was associated with increased homing of mast cells to, and/or increased proliferation within, tumor. However, given that mast cells in the control animals did not affect tumor progression, the pro-tumor function observed was shown to be specifically regulated by the microbiota. Given confirmation that antibiotic disruption of the gut microbiota has a detrimental effect on breast cancer, antibiotic-induced dysbiosis of the flora and dysregulation of the associated metabolites could be hypothesized to promote tumor growth by reprogramming mast cell homing and/or function. Future studies might consider determining the changes that occur in mast cells and breast tumor cells in response to gut dysbiosis. Kirkup et al. (147, 148) used a mixture of vancomycin, neomycin, metronidazole, and amphotericin (VNMA) to assess DNA concentrations in feces after microbiota dysbiosis and found very low DNA concentrations in the feces of the experimental group compared to the control group (water treatment). Importantly, the rate of PyMT-BO1 and EO771 breast tumor growth was significantly increased after disruption of the gut microbiota in the treated animals compared with the control animals (water treatment). Transcriptomic analysis also revealed dramatic differences in the regulation of metabolic pathways after antibiotic-induced dysbiosis of the intestinal flora, suggesting that accelerated breast cancer tumor growth might be associated with metabolic reprogramming. Fecal metabolomics was confirmed by 1H NMR spectroscopy analysis, which showed that 8 metabolites were elevated and 9 were significantly reduced in the major components of fecal samples from antibiotic-treated animals compared to fecal samples from control animals (147). Several of these amino acids (among them alanine, histidine and aspartic acid) were significantly increased in the antibiotic-treated animals. In contrast, the SCFAs butyrate and acetate, but not the branched-chain fatty acid isovalerate, were significantly reduced. Microbiota-derived butyrate is readily absorbed from the gut and can play a role in inhibiting histone deacetylases (153) in a variety of diseases, including cancer. Inhibition by butyrate can sensitize cancer cells to reactive oxygen species–induced apoptosis, thereby inhibiting the proliferation of breast cancer cells (154), but its role in the organism is yet to be confirmed in clinical trials. The authors hypothesized that a decrease in the bioavailability of the intestinal flora metabolite butyrate plays a role in enhanced tumor metabolism. Metabolites from the gut can reach distant tissues and organs such as the breast *via* the circulation, where they might play a role in regulating cancer cell function. Kirkup et al. noticed that antibiotics associated with breast cancer (e.g., cefadroxil, which is widely used in the USA after mastectomy). C57BL/6 mice carrying PyMT-BO1 tumor cells and receiving a cefadroxil dose equivalent to that in human patients experienced a significant acceleration in tumor growth. Analysis of the gut microbiota of the animals showed that the microbiota aggregates in samples from the experimental and control animals were independent and clustered differently before and after treatment. The relative abundance of *Lactobacillus* decreased over time in the control and experimental groups, and this appeared to be replaced mainly by fecal genera in the control animals, however, this did not occur in the antibiotic-treated animals. The genus with the most significant change in the microbial composition of the animals in the experimental group compared to the pre-treatment samples was *Lactobacillus*, but there was no significant difference before and after the control group. We presume that the disappearance of *Lactobacillus* might be driven by tumor cells, tumor–microbiota interactions, or natural maturation of the microbiome rather than by cefadroxil administration. Further analysis revealed differences in the abundance of 11 genera after cefadroxil treatment: *Mucispirillum*, *Marvinbryantia*, *Parabacteroides*, *Anaeroplasma*, *Bacteroides*, and *Paraprevotella* were significantly higher, and *Alloprevotella*, *Alistipes*, *Odoribacter*, *Faecalibaculum*, and *Anaerotruncus* were significantly lower. When multiple comparisons were made, 8 genera were significantly altered after antibiotic treatment. the genera that were significantly lesser abundant in treated animals relative to the controls, several are known butyrate-producing bacteria (e.g., *Odoribacter* and *Anaerotruncus*) or genera carrying the genes required for butyric acid production (e.g., *Faecalibaculum* and *Alistipes*) (147), consistent with the significant reduction in butyrate production observed in the metabolomic analysis of feces. That observation suggests that the use of a single antibiotic associated with breast cancer causes significant changes in microbiota genera and aggregation, potentially correlating with the tumor growth rate, but without a direct link to accelerated growth of breast tumors.

### 2.3 Effect of changes in metabolites following microbial perturbation on breast cancer metastasis

A major signaling route between the microbiome and the host is the secretion of Microbial metabolites that enter the circulation and reach their target cells (155–158). microbial metabolites synthesized in organs or glands (in this study, in the microbiome) function much like human hormones, in that they transfer to other anatomic locations and exert biologic effects (159). Microbial metabolites can enter the circulation and interfere with the steady-state of the intestinal and other local environments, acting as signaling mediators that influence the progression of breast cancer. SCFAs (160, 161), Lithocholic acid (LCA) (162–165), cadaverine (166), and de-conjugated estrogens (96, 109, 167), these metabolites have the ability to inhibit tumor-cell proliferation, the conversion of epithelial cells to mesenchymal cells, tumor metastasis, and cell migration and metastasis, and to induce antitumor immunity, to restructure cell metabolism, to induce senescence, and lower the number of tumor stem cells (164, 166, 168, 169).

The finding that perturbations in the gut microbiome are associated with tumor propagation at a distance supports the idea that the gut microbiome can be considered to be an endocrine gland (159, 170). Some metabolites associated with the activity of gut bacteria can enter the bloodstream and have been shown *in vitro* to affect the functioning of breast cancer and immune cells. Members of the microbiota can digest certain indigestible components of the human diet (e.g., dietary fiber), and SCFAs—for example, acetate, propionate, and butyrate—are components of metabolized dietary fiber (30, 171) and act as modulators of the host’s immune response. Bioactive compounds such as metabolic polyphenols (172) promote the growth of beneficial bacteria such as *Bifidobacterium* and *Lactobacillus* and produce SCFAs (173, 174). Some studies have shown that microbially derived homologous receptors for SCFAs were associated with a reduction in the invasive potential of breast cancer cells, with the homologous receptor FFAR2 inhibiting the Hippo-Yap pathway and increasing the expression of the adhesion protein E-cadherin, and FFAR3 inhibiting MAPK signalling (175), particularly butyrate, which has anticancer effects, as demonstrated in cancer cell cultures (176, 177) and animal models (160). Crucially, those microbial metabolites are produced after fermentation and/or metabolism of dietary components, and one of the key roles of the microbiota is to break down complex foods into simple bioactive compounds.

In the gut, disruption of the microbiota breaches the biologic barrier between it and the underlying tissue, leading to adverse physical contact between microbes and host cells, inducing paracrine production of bacterial metabolites (135). Changes in the microbiome have been associated with metabolic diseases such as obesity and type II diabetes (178), which are risk factors for certain cancers, including breast cancer (80, 179). The intestinal flora is responsible for the conversion of primary bile acids to secondary bile acids (180), and changes in the intestinal microbiota can therefore directly affect changes in secondary bile acids. Edit Mikóah et al. (169) studied three secondary bile acids—LCA, deoxycholic acid, and ursodeoxycholic acid. Of those three, LCA was found to exert a tumor-suppressive effect by reducing the growth of MCF7, SKBR3, and 4T1 breast cancer cells. They tested the cytostatic properties of LCA in mice transplanted with 4T1 breast cancer cells and found that the ability of the primary tumor to infiltrate surrounding tissues and metastasize was significantly reduced after LCA treatment. This study was the first to provide evidence for a mechanism of interaction between the microbiome and breast cancer by describing that LCA, a metabolite of microorganisms in the gut, is transferred to the breast *via* the bloodstream and might play an important role in promoting antiproliferative effects in breast cancer. However, LCA might be produced by the breast’s own microbiota and not only by the gut microbiota. The ratio of those two sources (breast and gut) in terms of LCA abundance is unknown and requires substantial research and continued trials.

Cadaverine is produced through lysine decarboxylation by lysine decarboxylase (181). *Shigella felis*, *Shigella sonnei*, *Escherichia coli*, and *Streptococcus* are all capable of expressing it (182). Kovács et al. (166) explored the effects of cadaverine supplementation (500 nmol/kg) on mice homozygously transplanted with 4T1 breast cancer cells and found a reduction in the aggressiveness of the primary tumor. Histologic examination of the primary tumors after cadaverine treatment showed a reduced mitotic rate and heterogeneity of nuclear morphology in the mammary tumor cells. To assess whether cadaverine treatment could convert mesenchymal-like carcinoma cells into epithelial-like cells, increased cadaverine resistance was measured using ECIS (Electric Cell-substrate Impedance Sensing), which showed better cell adhesion. To verify that finding, cells stained with Texas Red-X phalloidin and observed under microscopy showed that, after cadaverine treatment, the fibroblast-like morphology of 4T1, MDA-MB-231 and SKBR-3 breast cancer cells had changed to a cobblestone-like morphology that is characteristic of epithelial cells, and the inhibition of matrix metalloproteinase 9 expression also confirms the decrease in tumour cell migratory properties. A cellular flux analyser assessed the metabolic changes induced by necrotropism and found a reduction in glycolytic flux, which is characteristic of breast cancer mesenchymal cells (183). Cadaverine exerts its anticancer effects by inhibiting epithelial–mesenchymal transition, cell motility, chemotaxis, and metastasis. A further assessment of the “stemness” of 4T1 cells using an aldehyde dehydrogenase assay found that “stemness” was also slightly reduced (166). Dysbiosis of the intestinal flora (i.e., a change in the basal environment) leads to a change in the level and type of metabolites produced, which might have no effect on reducing the proportion of stem cells in breast cancer and slowing the rate of metastasis or might have the opposite effect, promoting malignant progression of the tumor. In the early stages of breast cancer in dysbiosis mice, bacterial cadaverine biosynthesis in the gut is reduced, leading to lower production of anti-cancer bacterial metabolites. We can speculate that in the presence of disturbed or slightly disturbed gastrointestinal flora, the metabolites produced act as signaling mediators and a specific crosstalk reaction may occur with the host, and this process may be directly or indirectly linked to the metastasis, migration and invasion of mammary tumors in mice.

### 2.4 Role of probiotics to block breast cancer spreading

A few studies have found that probiotic preparations are gaining in popularity for the improvement of health conditions such as antibiotic-induced diarrhea, irritable bowel syndrome, and obesity (184, 185). The use of probiotics can reduce or inhibit tumor growth, reduce tumor angiogenesis, tumor cell extravasation and lung metastasis (186). Long-term disturbance of the gut microbiome, which disrupts the probiotic structure and composition, may conversely increase the risk of breast cancer metastasis (187, 188).

**Figure 1:**
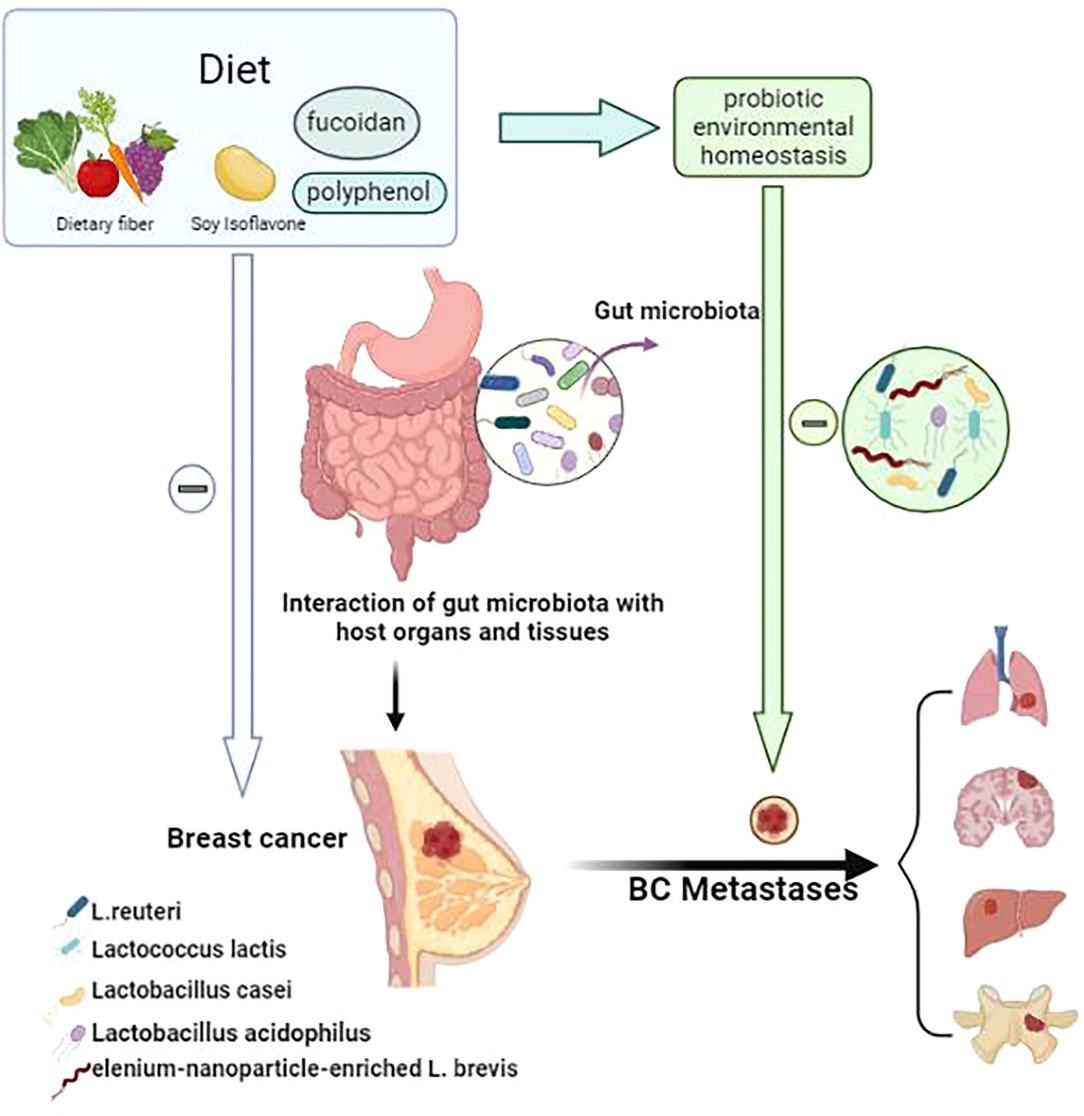
The linkage between probiotic environmental homeostasis and breast cancer metastasis. A good diet (e.g. foods rich in dietary fiber, soy isoflavones, fucoxanthin and polyphenols) can reduce intestinal flora dysbiosis and thus harmonize the body to reduce the incidence and metastasis of breast cancer. Diet as an important factor in the stable composition of the host probiotic environment, through intestinal flora regulation. probiotic environmental homeostasis can play an adjuvant anti-cancer role in the progression and metastasis of breast cancer (lung, brain, liver, bone).

*Lactobacillus casei*, a type of probiotic, is a Gram-positive bacterium that is resistant to the body’s defense mechanisms. After entering the human body, *L. casei* can survive in large numbers in the intestinal tract and can play a role in regulating the balance of intestinal flora, promoting digestion and absorption, among other processes (189). It is highly effective in lowering blood pressure (190) and cholesterol (191), promoting cell division and antibody immunity, enhancing human immunity, preventing cancer, and inhibiting tumor growth. Aragón et al. (186) used milk fermented with *L. casei* CRL431 to evaluate its possible effects on tumor growth, tumor cell extravasation and lung metastasis in a mouse model. By comparing mice fed fermented milk (FM), mice fed regular milk and mice not fed any special food, it was found that the group fed FM showed an inhibition of tumor growth and a decrease in tumor vascular filling, tumor cell extravasation and lung metastasis. Khoury et al. (192) used kefir water, a fermented milk product containing probiotics, to treat BALB/c mice that had been transplanted with 4T1 mammary cancer cells and, in the treated mice, detected a significant reduction in tumor size and weight, a significant enhancement of helper T cells and cytotoxic T cells, a significant reduction in lung and bone marrow metastases. Zamberi et al. (193) found that kefir water (mix of *Lactobacillus acidophilus*, *Lactobacillus casei*, and *Lactococcus lactis*) exerted an anti- angiogenic effect on mouse mammary tumors by down-regulating the tumor-promoting invasive interleukin 1β and vascular endothelial growth factor (a key mediator of angiogenesis). In the above model, levels of the pro-angiogenic factor interleukin 6 were found to have declined (186, 189, 194, 195) after probiotic treatment, suggesting that downregulation by Lactobacillus might affect the metastatic potential of cancer cells. some study (186, 196, 197) demonstrated that milk fermented with *Lactobacillus casei* CRL431 (probiotic fermented milk (PFM)) reduced the side effects of capecitabine and reduced intestinal mucositis and mortality in a mouse model of breast cancer by modulating the immune response, this suggests the potential of PFM as a probiotic as an immune adjuvant that may reduce tumor growth and metastasis without compromising the anti-tumor/anti-metastatic effects of chemotherapy. They differentially regulate cancer-related signaling pathways in a cell-type-specific manner and play a suppressive role in the pro-tumor microenvironment (198–200). Conversely, disruptions in the intestinal flora might simultaneously or subsequently affect the probiotic environment, which could cause probiotics to lose their “dominant” role in the tumor environment, negatively affecting the control or inhibition of breast tumor cell growth or even accelerating the growth of tumor cells and promoting angiogenesis, becoming an indirect contributor to tumor metastasis. Yazdi et al. (201) demonstrated that selenium-nanoparticle-enriched L. brevis administered to mammary tumor-bearing BALB/c mice induced an effective immune response, resulting in reduced liver metastases and an increased lifespan, included increases in the T helper cytokines, interferon-gamma and interleukin 17, and enhanced natural killer cell activity. Hassan Z et al. Demonstrated that (202) *Enterococcus faecalis* and *Staphylococcus hominis* can significantly inhibit cell proliferation, induce apoptosis, and cell cycle arrest, and that they have no cytotoxic effect on normal cells, making them a good alternative drug for breast cancer treatment (**Figure 1**).

**Figure 1**. The linkage between probiotic environmental homeostasis and breast cancer metastasis

Probiotics have specific anticancer properties, and studies have shown that they can alter the expression of various genes involved in apoptosis (203), invasion and metastasis (204), maintenance of cancer stem cells (205), and control of the cell cycle (206). Probiotics have been highlighted as superior in the treatment of cancer. however, more pre-clinical and clinical studies are needed to determine which strains are beneficial during specific treatments before probiotic administration is considered safe and customisable for all individuals.

### 2.5 Diet affects the likelihood of breast cancer progression

Although the correlations between BRCA risk and dietary intake have been intensively studied, the underlying associations or effector mechanisms remain poorly understood. Historically, increased risk of BRCA has been tied to high intake of red meat and animal fat (207, 208), with decreased risk being concurrently linked to fruit and vegetables consumption (209). Changing dietary patterns affects the microbiome and Indirect affects the development of breast cancer. A case-control study in Japan showed that regular consumption of Lactobacillus casei Shirota and soy isoflavones from puberty onwards reduced the incidence of breast cancer in Japanese women (210); Newman TM et al. also indicated that the Mediterranean diet could prevent breast cancer, because of its inclusion of an abundance of plant-based foods and the lack of processed foods (211). Xue M et al. confirmed through experiments (212) that fucoidan increases the diversity of intestinal flora and can promote the intestinal barrier function, and he suggested fucoidan as a preventive agent for breast cancer. studies have shown that increased polyphenol intake is associated with higher levels of beneficial bacteria (such as *Bifidobacterium* and *Lactobacillus*) and SCFAs in humans (174), while also decreasing levels of bacteria that have been associated with disease, so‐called pathobionts. Diet is an important factor in all microbiota studies and can help maintain the stability of gut microbes, which can influence the development of breast cancer. If dietary interventions are to be successfully used in future treatments, studies of diet and microbiota metabolites might have to be conducted in parallel. Indeed, recent studies have highlighted the personalised response to individuals (and the microbiota) to the same diet (213), which highlights the limitations and challenges for next‐stage studies of this kind.

Alcohol consumption increases the risk of breast cancer, although alcohol itself is not a direct carcinogen, acetaldehyde, a product of alcohol metabolism, is a mutagen which can form adducts with protein and DNA, inducing gene mutation, DNA crosslinks and chromosomal aberrations (214–216). Many studies have also confirmed that alcohol consumption not only induces breast cancer development (217, 218) but also promotes the progression of existing breast tumors and induces a more aggressive phenotype (219–222). There are no clear reports to confirm the correlation between alcohol, microorganisms and breast cancer metastasis, but there is no doubt that alcohol causes dysbiosis of the intestinal flora (223–225). It is not difficult to guess that there may be an alcohol-gut flora-breast cancer axis, which means that changing lifestyle habits could have profound implications for the prevention and prognosis of the disease, but the role of gut flora in this needs to be studied in depth.

Details of the following studies included in this review are summarized in **Table 3**.

**Table 3:** Correlation between factors that disturb the intestinal flora and breast cancer metastasis and progression.

Factor	Study model	Regulation			Key biologic function
ETBF					
BFT	BALB/C, MCF-7, MCF-10A		Up	ETBF produces BFT, which is highly invasive in breast cells and expresses migration- and metastasis-related genes (137–139)	
Antibiotics					
	MMTV-PyMT		Up	Dysbiosis was associated with enhanced distant metastasis and dissemination of breast tumor cells (140)	
	BRPKP110		Up	Infiltration of myeloid cells in breast tissue was enhanced after FMT perfusion (140)	
	GFP+ tumor cells		Up	Dysbiosis was associated with increased breast tumor cell dissemination (140)	
M2-like macrophages			Up	Dysbiosis was associated with enhanced infiltration of myeloid cells into the breast tissue (140, 141)	
VNMAA or vancomycin	PyMT-BO1		Up	Significant reduction in gut microbiota abundance and accelerated tumor growth were observed after VNMAA treatment (146)	
	E0771		Uncertain	Increased homing/value-added of mast cells in breast cancer tumors where gut microbiota were disturbed after antibiotic treatment (146)	
VNMA	PyMT-BO1, E0771		Up	Antibiotic-induced dysbiosis of microflora was associated with reduced expression of pro-apoptotic genes and increased expression of pro-survival genes (153)	
	PyMT-BO1, E0771		Up	Antibiotic administration was associated with dramatic differences in the regulation of microbial metabolic pathways and increased tumor growth rates in laboratory animals (146, 148)	
cefadroxil	PyMT-BO1		Up	Gut microbial aggregation, genus differences, and accelerated tumor growth were observed in cefadroxil-treated animals (146, 148)	
Probiotics					
CRL431			Down	FM was associated with inhibited mammary tumor growth and metastasis in mice (185)	
Kefir water	4T1, BALB/C		Down	Administration of kefir water was associated with inhibition of tumor size and distant metastasis with downregulatory effect (190, 191)	
*L. brevis*	BALB/C		Down	*L. brevis* administration was associated with immune response and reduced liver metastases from mammary carcinoma in mice (195)	
Microbial metabolite					
SCFAs	SCFAs		Down	Butyrate has anti-cancer properties (159, 175, 176)	
LCA	MCF-7, SKBR3, 4T1		Down	LCA was associated with inhibition in the growth of breast cancer cells (180) and reduction in infiltration by the primary tumor into the surrounding tissue and metastasis (168)	
Cadaverine	4T1, MDA-MB-231, SKBR3		Down	Cadaverine can fight breast cancer progression by inhibiting EMT, cell motility, chemotaxis, and metastasi (182)	
Diet					
*Lactobacillus casei Shirota* and Soy isoflavones from puberty onwards (207)			Uncertain		
polyphenol (173)			Uncertain		
Fucoidan (209)			Uncertain		

ETBF, Enterotoxigenic Bacteroides fragilis; BFT, B. fragilis; BALB/C, experimental mouse; MCF-7, human breast cancer cells; MCF-10A, epithelial cell line; MMTV-PyMT, mouse model of highly metastatic breast cancer; BRPKP110, HR+ mouse breast cancer cell line model; FMT, fecal microbiota transplantation; GFP+, Green fluorescent protein; VNMAA, vancomycin, neomycin, metronidazole, amphotericin, ampicillin; PyMT-BO1, PyMT-derived ductal cell line *in situ* mammary fat pad injection model; E0771, spontaneously derived basal cells; VNMA, vancomycin, neomycin, metronidazole, amphotericin; FM, fermented milk; CRL431, type of L. casei used to ferment milk; 4T1, breast cancer cells; SCFAs, short-chain fatty acids; TLR4, Toll-like receptor 4;LCA, lithophanic acid; MDA-MB-231, breast cancer cells; SKBR3, breast cancer cells; EMT, epithelial–mesenchymal transition.

## 3 Conclusions and future prospects

Globally, the number of factors affecting gastrointestinal dysbiosis is increasing and the gastrointestinal microbiome is emerging as an important player in the risk and progression of breast cancer. This provides an exciting new perspective on breast cancer metastasis, namely that the causes of intestinal dysbiosis are complex and variable, and that there may be a complex causal relationship between progression and metastasis of breast cancer. Therefore, treating the gut flora to stabilize the microenvironment may reduce pro-tumorigenic factors and their propagation in the tissue microenvironment, and establishing new strategies to balance these deleterious fluctuations is of interest in the treatment and prognosis of breast cancer. Given that several intrinsic and extrinsic factors are known and that the gut microbiota and breast cancer have an interactive relationship, future sequencing of the microbiota to capture metadata about dysbiosis and the selection of *in vivo* models are expected. Those steps will be informative and positive in reducing the risk of breast cancer progression and metastasis, and in guiding therapy for gastrointestinal symptoms or prognosis in patients with breast cancer. Future studies analyzing the gastrointestinal microbiota in patients with breast cancer should consider definitive stratification by histology and molecular science, which could require longer experience and a longer time frame. In addition, because of the large number of complex resident gut flora species, the difficulty of data collection and the unclear specific mechanisms of microenvironmental changes due to dysbiosis, studies and evidence linking the gastrointestinal microbiota to breast cancer metastasis and progression are currently relatively scarce and need to be validated by more specific and high-quality clinical trials and data, and there is an urgent need to combine different disciplines and microbiome studies and design new technical approaches.

## Data availability statement

The current state of research and references in this article (review) are cited from the relevant references and the data are authentic and publicly available.

## Author contributions

JZ, ZL, MD, XH and MY contributed to the conception and the drafting of manuscripts. GS, QX and YZ are responsible for coordinating and participating in the article revision. All authors contributed to the article and approved the submitted version.

## Funding

The project was supported by the 2022 Qinghai Province Central Guide to Local Science and Technology Development Fund, the Breast Disease Treatment Centre of the Affiliated Hospital of Qinghai University manages this funding.

## Conflict of interest

The authors declare that the research was conducted in the absence of any commercial or financial relationships that could be construed as a potential conflict of interest.

## Publisher’s note

All claims expressed in this article are solely those of the authors and do not necessarily represent those of their affiliated organizations, or those of the publisher, the editors and the reviewers. Any product that may be evaluated in this article, or claim that may be made by its manufacturer, is not guaranteed or endorsed by the publisher.

## Abbreviations

TNBC, Triple‐negative breast tumors; LPS, Lipopolysaccharide; iNKT, Invariant natural killer T; GLP-1, glucagon-like peptide-1; BBB, blood-brain barrier; BC, Breast cancer; VNMA, vancomycin, neomycin, metronidazole, and amphotericin; SCFAs, short-chain fatty acids; LCA, lithophanic acid; HR+, hormone receptor–positive; FM, fermented milk; FMT, fecal microbiota transplantation; ETBF, enterotoxigenic B. Fragilis; BFT, B. fragilis toxin.
